# Angioedema Secondary to Tenecteplase Use in a Patient with Acute Ischemic Stroke: A Case Report

**DOI:** 10.5811/cpcem.7190

**Published:** 2024-07-11

**Authors:** Babette Newman, Matthew Poremba, R. Gentry Wilkerson

**Affiliations:** * University of Maryland Medical Center, Department of Emergency Medicine, Baltimore, Maryland; † University of Maryland Medical Center, Department of Pharmacy, Baltimore, Maryland

**Keywords:** *angioedema*, *tenecteplase*, *stroke*, *airway*

## Abstract

**Introduction:**

Angioedema, a swelling of the subcutaneous or submucosal layers of the skin or gastrointestinal tract, is a potential complication to thrombolytic therapy in the treatment of acute ischemic strokes. In these cases, angioedema develops due to increased levels of bradykinin as a result of the activation of the fibrinolytic pathway and contact activation system. Angioedema can involve the tongue, larynx, and vocal cords, leading to occlusion of the airway and death due to asphyxiation. It is vital for the emergency physician to know that this complication can occur to ensure appropriate monitoring for development of angioedema.

**Case Report:**

We report the case of a 65-year-old Black man who presented with signs of an acute ischemic stroke and was treated with tenecteplase. The patient’s stroke symptoms mostly resolved within 90 minutes; however, he developed swelling of his right upper lip consistent with angioedema. The patient was treated with steroids and antihistamines. He was closely monitored and did not require airway intervention. The angioedema was almost fully resolved by the following day.

**Conclusion:**

Angioedema is a known complication of thrombolytic therapy for acute ischemic stroke. Risk factors for alteplase-associated angioedema include use of angiotensin-converting enzyme inhibitors, female gender, diabetes, and infarcts of the insula and frontal cortex. As hospital systems switch from alteplase to tenecteplase for the treatment of acute ischemic strokes for reasons of cost and ease of administration, it is important to recognize that angioedema is also a potential complication of tenecteplase.

Population Health Research Capsule What do we already know about this clinical entity?

*Bradykinin-mediated angioedema occurs due to C1-esterase inhibitor deficiency, angiotensin-converting enzyme (ACE) inhibitors, and thrombolytics.*

What makes this presentation of disease reportable?

*There are few reports of angioedema due to tenecteplase. This is the first description involving a patient not on an ACE inhibitor.*

What is the major learning point?

*Clinicians should be aware of angioedema as a potential complication of tenecteplase use. It occurs in about 1.1% of patients receiving tenecteplase.*

How might this improve emergency medicine practice?

*This report underscores the need for close patient monitoring after giving tenecteplase, as the patient’s airway may become compromised due to angioedema.*

## INTRODUCTION

Angioedema describes the development of swelling of the subcutaneous or submucosal layers of the cutaneous and gastrointestinal tracts. Numerous mechanisms can result in the development of angioedema, and these are usually classified based on the underlying substance causing the swelling. Histaminergic angioedema is due to the release of histamine and is seen in cases of allergy and anaphylaxis as well as other immunologic processes. Bradykinin-mediated angioedema includes hereditary angioedema (HAE), which is usually caused by deficient quantity or function of C1-esterase inhibitor (C1-INH), acquired angioedema where the levels of C1-INH are low due to increased consumption, and medication-associated increases in bradykinin including angiotensin-converting enzyme (ACE) inhibitors.^1^ In ACE inhibitor-induced angioedema, the ACE enzyme, which also acts as the primary enzyme to break down bradykinin, is inhibited leading to increased levels of bradykinin.^2^

Numerous case reports have described the development of angioedema following administration of alteplase for the treatment of acute ischemic stroke. In many of these cases the swelling is contralateral to the infarcted hemisphere.^3^ When plasminogen is activated to plasmin, the plasmin activates Factor XII to Factor XIIa, which in turn converts prekallikrein to kallikrein, the enzyme responsible for the cleavage of bradykinin from high molecular weight kininogen.^1^ Herein we describe a case of angioedema that developed after administration of tenecteplase (TNK). Individual reports of angioedema secondary to tenecteplase have rarely been described,^4^ and to date none have been in a patient not taking an ACE inhibitor.

## CASE REPORT

A 65-year-old man presented to the emergency department (ED) via emergency medical services as a stroke alert for acute onset right-sided facial droop, dysarthria, and aphasia. The patient was reportedly found outside by a neighbor who noticed that he was having trouble speaking. On arrival, the patient’s neighbor and family members could not be reached for additional information, but his last known well time was estimated to be approximately one hour prior to arrival in the ED. The patient denied any preceding head trauma as well as associated headache, dizziness, vision changes, chest pain, shortness of breath, nausea, or vomiting. His past medical history included hypertension, well-controlled HIV, and previous successful treatment for hepatitis C. He had no history of prior strokes and was not taking anticoagulants. He had documented allergies to lisinopril and shellfish. The patient’s initial vital signs were notable for hypertension to 156/84 millimeters of mercury (mm Hg), but otherwise were within normal limits with heart rate 96 beats per minute, respiratory rate 18 respirations per minute, pulse oximetry 96% on room air, and temperature 98.4° Fahrenheit.

On physical exam, the patient was alert and interactive. His speech was moderately dysarthric and aphasic but intelligible with effort. He had facial paralysis on the right. He was able to answer one question correctly and follow two commands correctly. There was no gaze deviation, visual field loss, extremity drift, sensory loss, limb ataxia, or extinction. His initial National Institutes of Health Stroke Scale score was five. The brain attack team was called, and the patient was quickly taken for computed tomography (CT) of the head as well as CT angiography of the head and neck with perfusion. Imaging revealed no intracranial hemorrhage or large vessel occlusion. Since the patient presented within 4.5 hours of symptom onset and there were no obvious contraindications, the decision was made to administer TNK with emergency consent.

The patient received an intravenous (IV) 0.25 milligrams per kilogram (mg/kg) bolus of TNK, 16 mg total. Shortly after TNK administration, his blood pressure increased to 188 mm Hg, so he was started on a clevidipine infusion at 1 mg/hour. Approximately 90 minutes after TNK administration, the patient’s neurologic deficits had improved, but he was found to have developed profound swelling of his right upper lip, consistent with angioedema (Image). There was no associated tongue swelling, drooling, or stridor. He had no increased work of breathing and continued to saturate well at 95% on room air. The patient was treated with IV methylprednisolone 125 mg, famotidine 20 mg, and diphenhydramine 50 mg, and close monitoring was continued in the ED. He was ultimately admitted to the neurologic intensive care unit where the angioedema was noted to be almost fully resolved by the following day. Magnetic resonance imaging was performed the following day demonstrating a distal left middle cerebral artery infarct.

**Image.**
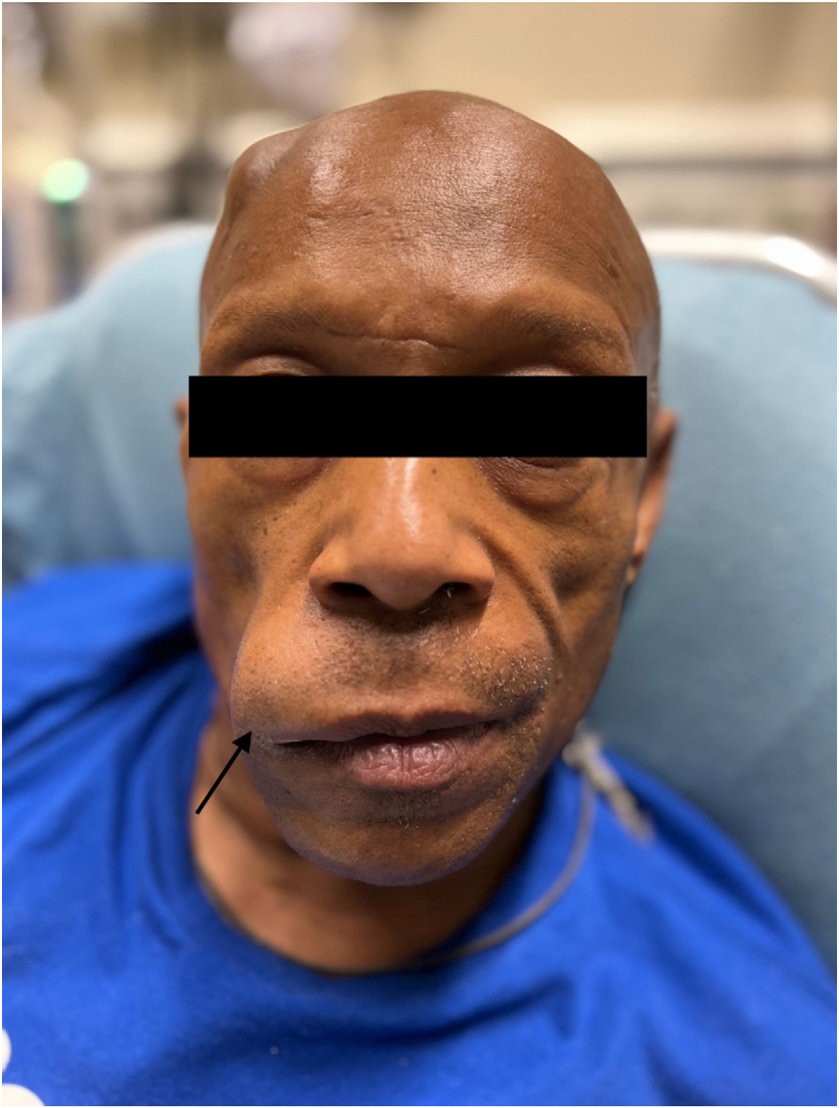
Angioedema of the right upper lip in a patient following administration of tenecteplase (arrow).

## DISCUSSION

Alteplase is the current standard of care treatment for acute ischemic stroke and is the only US Food and Drug Administration-approved agent for this indication. Tenecteplase is considered an alternative to alteplase in the 2019 American Heart Association/American Stroke Association guidelines.^5^ It is a genetically modified alteplase variant with increased fibrin specificity and a longer half-life. These modifications allow TNK to be given intravenously as a single, rapid bolus dose, whereas alteplase requires a bolus dose followed by a one-hour infusion.^6^ Recently, multiple trials comparing TNK with alteplase for treatment of stroke have shown TNK to be not inferior in both efficacy and safety.^7^ ^,^ ^8^ Data from these trials, in combination with the ease of administration and improved cost effectiveness, has prompted many health systems to switch from alteplase to TNK for stroke treatment.^2^ ^,^ ^9^

Published studies comparing TNK and alteplase for treatment of stroke most commonly report symptomatic intracranial hemorrhage or any intracranial hemorrhage as their primary safety endpoints, while rates of angioedema are reported inconsistently. The largest published trial comparing alteplase to TNK reported that 9/800 patients (1.1%) receiving TNK and 9/763 patients (1.2%) receiving alteplase experienced angioedema.^3^ In a single-center retrospective analysis of patients treated with alteplase, the rate of angioedema was reported to be 7.9%.^10^ Rose et al performed a systematic review and meta-analysis of complications of TNK and alteplase for stroke and found cumulative angioedema rates of 0.56% in patients with TNK compared to 0.63% in patients receiving alteplase.^11^

Overall rates of angioedema appear to be similar between alteplase and TNK, but specific characteristics of patients who experience angioedema are seldom reported. A 2022 meta-analysis from Mas Serrano et al found prior treatment with ACE inhibitors, diabetes, dyslipidemia, female gender, and hypertension to be associated with development of angioedema after alteplase administration.^12^ Strokes involving the frontal cortex and insula have also been described as having increased risk of angioedema.^3^ Potential risk factors for experiencing angioedema after TNK administration are not well described in current literature, although they are expected to be similar to those seen with alteplase given both agents share their mechanism of action. The only published case reports of angioedema after TNK administration occurred in a patients who were currently using an ACE inhibitor, which is a known risk factor for development of angioedema.^4^ ^,^ ^13^ Our patient was not currently taking an ACE inhibitor at the time he received TNK, although he had documented history of an allergic reaction to the ACE inhibitor lisinopril.

There have been no studies to determine the best treatment of angioedema following TNK administration. The primary goal is to ensure airway patency. Despite histamine not being the substrate causing angioedema following TNK administration, some have suggested that it should be treated similarly to allergic reactions by administering antihistamines, steroids, and in some cases epinephrine. The use of medications that are approved for HAE have not been studied and are currently not recommended for this indication. The lysine analog, tranexamic acid (TXA), could have a potential benefit as it binds to plasmin, preventing the activation of Factor XII leading to decreased production of bradykinin. Tranexamic acid is an antifibrinolytic in that it prevents plasmin degradation of fibrin-to-fibrin degradation products. Use of TXA may lead to worse outcomes in patients with acute ischemic strokes.

## CONCLUSION

This case report provides details regarding the development of angioedema in a patient being treated with tenecteplase for an acute ischemic stroke. While it is known that angioedema is a potential complication of TNK administration, the details of individual cases are rarely reported. It is important for the emergency physician to be aware of this potential complication of TNK so that patients do not experience further morbidity and mortality.
